# Supplementary material for: Analysis of Expression of the ANG1, CaSR and FAK Proteins in Uterine Fibroids
Source: Int J Mol Sci. 2024 Jun 28;25(13):7164. doi: 10.3390/ijms25137164 (PMC11241732; doi:10.3390/ijms25137164)
Supplement: Supplementary file 1 [file ijms-25-07164-s001.zip › ijms-2984462-supplementary.pdf]

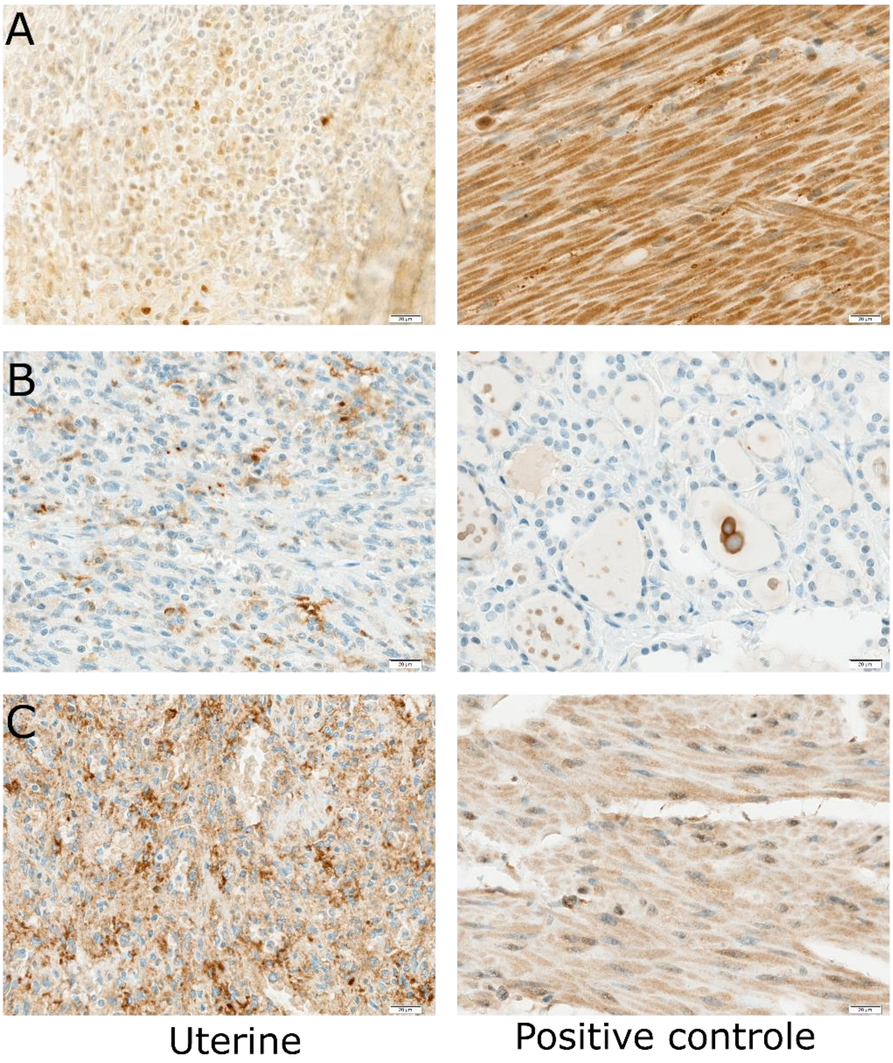

Supplementary Figure 1. Immunohistochemical expression of the analyzed protein in the uterine of the same patients: ANG1 (A), CaSR (B), FAK (C). Magnification for all pictures is 400X.
